# Long-Term Quality of Life after Endoscopic Pituitary Adenoma Surgery with Nasoseptal Flap Reconstruction

**DOI:** 10.5041/RMMJ.10435

**Published:** 2021-04-29

**Authors:** Shadi Shinnawi, Ilya Kopaev, Shorook Na’ara, Ayelet Eran, Gil Sviri, Dmitry Ostrovsky, Ziv Gil

**Affiliations:** 1 Department of Otolaryngology-Head and Neck Surgery, Rambam Health Care Campus, Haifa, Israel; 2 The Laboratory for Applied Cancer Research, Rambam Health Care Campus, Haifa, Israel; 3 Department of Radiology, Rambam Health Care Campus, Haifa, Israel; 4 Department of Neurosurgery, Rambam Health Care Campus, Haifa, Israel

**Keywords:** Endoscopic pituitary adenoma surgery, nasoseptal flap reconstruction, quality of life

## Abstract

**Introduction:**

Endoscopic endonasal transsphenoidal surgery (EETS) on the pituitary gland is considered safe and efficacious. The nasoseptal flap (NSF) is sometimes used to prevent or repair postoperative cerebrospinal fluid (CSF) leaks. Few investigators have quantified long-term quality-of-life (QOL) outcomes regarding sinonasal measures after EETS, with or without involvement of the NSF. This study assesses whether the septal flap affects sinonasal QOL outcomes for patients receiving EETS for pituitary adenoma.

**Methods and Materials:**

This is a retrospective study of patients who underwent EETS between 2013 and 2018. A total of 62 adults completed the Sinonasal Outcome Test-22 (SNOT-22) at least one year after the surgery. Outcome measures were compared between patients who underwent EETS with and without septal flap reconstruction.

**Results:**

For the entire cohort, there were 14 patients (22.6%) who had septal flap reconstruction and 48 patients (77.4%) who did not. Patient demographics, tumor characteristics, surgical outcomes, and duration between surgery and completion of the questionnaire were similar for both groups. The mean SNOT-22 scores in the no reconstruction (NR) group and the nasoseptal flap reconstruction (NSFR) group were similar (*P*=0.9). In terms of SNOT-22 subdomains (rhinologic symptoms, extranasal rhinologic symptoms, ear/facial symptoms, psychological dysfunction, and sleep dysfunction), no significant differences were found when comparing the groups.

**Conclusion:**

As compared with no reconstructive involvement, NSF utilization does not affect the QOL and nasal symptoms of patients undergoing EETS.

## INTRODUCTION

Over the last century, pituitary adenoma surgery has evolved from transcranial approaches to less invasive transsphenoidal approaches.^1^ Modern endoscopic pituitary surgery was introduced in France in 1992 and in the United States in 1997.^2^,^3^ In more recent years, this endoscopic technique has become widely accepted by otolaryngologists and neurosurgeons around the world, and its efficacy, safety, advantages, and disadvantages have been evaluated in numerous studies.^4^–^10^

The nasoseptal flap (NSF) is a neurovascularized mucoperichondrial and mucoperiosteal axial pattern flap, which is situated on the posterior branch of the sphenopalatine artery.^11^ It is used commonly as part of the reconstructive phase of endoscopic pituitary surgery, primarily to prevent and/or seal cerebrospinal fluid (CSF) leaks, as well as to reconstruct the surgical defect to provide a healthy nasal microenvironment.^12^ However, the impact of the nasoseptal flap reconstruction (NSFR) on nasal function due to manipulation of nasal mucosa has been a major concern, as nasal complications such as crusting, septal perforation, and cartilage necrosis have accompanied endoscopic endonasal approaches and NSF utilization.^13^,^14^ These complications and their impacts on quality of life (QOL) have been the subject of investigations by several authors.^15^–^20^

The Sinonasal Outcome Test-22 (SNOT-22) questionnaire is a validated, patient self-assessment tool, which measures symptom severity and health-related QOL issues as they relate to sinonasal conditions.^21^,^22^ Although not specifically designed for this purpose, SNOT-22 has been used in several recent studies to evaluate the impact of endoscopic endonasal skull base approaches on the QOL of patients with skull base pathologies.^15^,^16^,^23^,^24^ However, to date, little has been published on long-term QOL outcomes, specifically sinonasal measures following endoscopic endonasal transsphenoidal surgery (EETS). Accordingly, in this study, we focus on comparing postoperative SNOT-22 QOL measures between patients who underwent EETS for pituitary adenoma with NSFR and those who underwent the surgery with no reconstruction.

## MATERIALS AND METHODS

The study is based on a review of the hospital charts of and questionnaire responses from patients that underwent EETS during the years 2013–2018, and who had pathology reports compatible with pituitary adenoma. All surgeries were performed by the same interdisciplinary team at the Rambam Health Care Campus in Haifa, Israel.

Eligible patients for study inclusion were over 18 years of age who underwent EETS for pituitary adenoma at least 12 months prior to the study. Exclusion criteria were as follows: (1) the existence of other skull base lesions; (2) pre-existing sinus disease; (3) nasal allergies; (4) intranasal drug abuse; (5) subjective olfactory disturbance at baseline; or (6) previous transsphenoidal pituitary surgery.

### Questionnaire

Patients were surveyed a single time, postoperatively, via mobile phone using the SNOT-22, which is an adaptation of prior, disease-specific instruments that have been validated in the otolaryngology literature.^21^,^25^–^27^ The SNOT-22 contains 22 items divided into five domains (rhinologic symptoms, extranasal rhinologic symptoms, ear/facial symptoms, psychological dysfunction, and sleep dysfunction).^28^ Items are scored on a 0–5 scale, where 0 reflects “no problems” and 5 indicates a “problem as bad as it can be.” Total scores can range from 0 to 110, with higher scores indicating worse QOL.

### Surgical Approach

Preparation and initial steps are critical to the success of NSF harvesting. Prior to elevation of the NSF, topical and local decongestants are used. Bilateral out-fracturing of inferior turbinates are carried out, and the sphenoid ostium is exposed. After inspection of the nasal cavity, the middle turbinate on one side is resected in its caudal part. At this stage, the NSF is elevated. With the use of a scalpel, the first incision is performed along the junction of the lower border of the nasal septum from posterior to anterior. Posteriorly, the incision reaches the choana and curves along its superior edge toward the medial maxillary wall. Superiorly, the incision reaches below the sphenoid ostium and curves superior to the sphenopalatine artery. Anteriorly, the incisions should reach the limen nasi. The edges of the flap should be right angled to achieve maximal coverage of the flap. Next, using a Freer retractor, the flap is meticulously elevated laterally up to the level of the sphenopalatine foramen. It is then stored in the choana or in the maxillary antrum.^29^

### Ethical Considerations

The study was conducted after its protocol was approved by the Helsinki Committee review board at our institution.

### Statistical Analysis

The results are presented as mean±SD for quantitative variables and number for categorical variables. Fisher’s exact test and *t* test were applied for comparison of categorical and quantitative variables, respectively. All statistical assessments were 2-sided and evaluated at the 0.05 level of significant difference, using IBM SPSS Statistics 22 for Mac (IBM Corporation, Armonk, New York, USA).

## RESULTS

Ninety-one patients were eligible for enrollment in this study. The response rate for completing the questionnaire—after excluding those patients who died, who were lost to follow-up, or who were operated on <12 months before the study began—was 68.1%. Thus, 62 patients participated (50% male and 50% female; ranging in age from 19 to 81 years, with a mean age of 51.58±15.16 years), completing the SNOT-22 ≥1 year postoperatively.

Patients were divided into two groups according to the use or non-use of NSF for reconstruction of the skull base defect: 14 patients in the NSFR group, and 48 patients in the no reconstruction (NR) group. Among the former, 3 patients underwent the surgery less than 24 months before answering the questionnaire; among the latter, 16 patients underwent the surgery less than 24 months before answering the questionnaire, *P*=0.51. As shown in Table 1, demographic and clinical characteristics were similar between the groups.

**Table 1:** Demographic and Medical Characteristics of the 62 Patients.

Characteristics	No. (%) of Patients		*P* Value

	NSFR Group (*n*=14)	NR Group (*n*=48)	
Sex			.36
Female	9 (64)	22 (46)	
Male	5 (36)	26 (54)	

Age, mean±SD, y	52.35±15.82	51.35±15.12	.82

Time from surgery, mo			.51
<24	3 (21)	16 (33)	
≥24	11 (79)	32 (67)	

Comorbidity			.99
Yes	5 (36)	16 (33)	
No	9 (64)	32 (67)	

Tumor size, mean±SD, mm	16.92±4.22	19.17±7.34	.27

NR, no reconstruction; NSFR, nasoseptal flap reconstruction; SD, standard deviation.

All 62 patients underwent successful removal of a pituitary adenoma via the transsphenoidal endoscopic approach. Mean hospitalization time was 4.2±1.3 days, with no significant difference between the groups. There were no postoperative mortalities and no documented cases of CSF leakage, meningitis, tension pneumocephalus, or disease recurrence.

**Figure 1:**
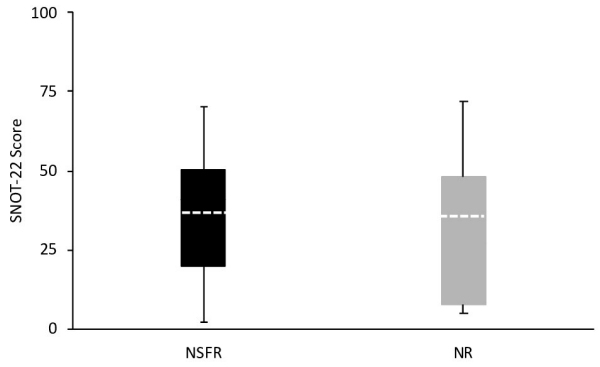
Comparison of Overall Sinonasal Outcome Test (SNOT-22) Scores Between the Two Surgical Groups The horizontal dotted lines within the boxes represent the mean SNOT-22 score; the bottom and top lines of the boxes, the 25th and 75th percentiles; and the whiskers extending below and above the boxes, the minimum of the 25th percentile and the maximum of the 75th percentile, respectively.

**Figure 2:**
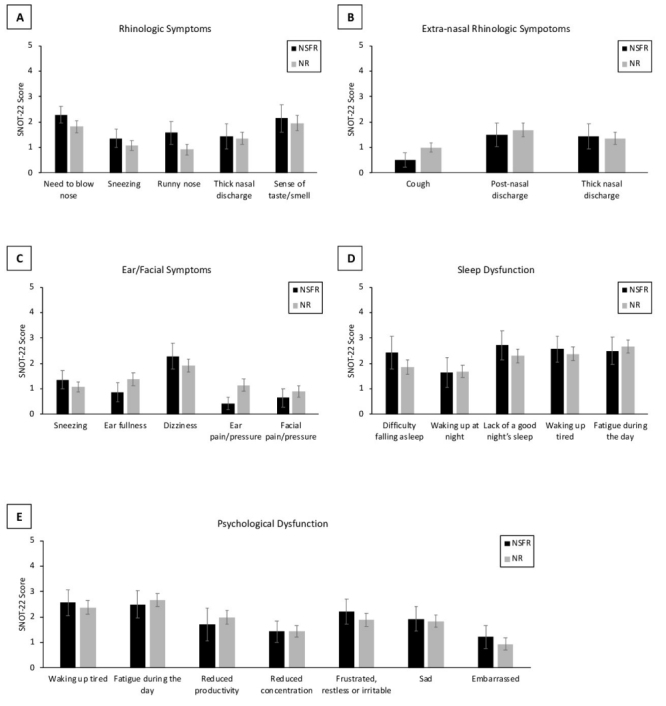
Comparison of Sinonasal Outcome Test (SNOT-22) Subdomain Scores Between the Two Surgical Groups **A:** Rhinologic symptoms. **B:** Extra-nasal rhinologic symptoms. **C:** Ear/facial symptoms. **D:** Sleep dysfunction. **E:** Psychological dysfunction. Error bars indicate standard error.

We first compared the mean overall postoperative SNOT-22 scores according to surgical approach employed. Patients in the NR group reported similar scores compared to the NSFR group (35.97±21.47 and 36.78±22.17, respectively), *P*=0.9 (Figure 1). Next, in assessing the postoperative scores of subdomains, we found that for each there were no statistically significant differences between the groups (Table 2). Finally, in evaluating each of the SNOT-22 questionnaire items separately, we found that no significant differences existed between the groups (Figure 2).

**Table 2:** Postoperative (≥12 months) Mean Sinonasal Outcome Test (SNOT-22) Subdomain Scores.

Subdomain	Mean±SD		*P* Value
	NSFR Group (*n*=14)	NR Group (*n*=48)	
Rhinologic symptoms	10.21±0.41	9.00±0.44	0.55
Extranasal rhinologic symptoms	3.43±0.56	4.04±0.34	0.54
Ear/facial symptoms	5.57±0.74	6.42±0.40	0.62
Psychological dysfunction	13.57±0.52	13.13±0.57	0.88
Sleep dysfunction	11.86±0.42	10.88±0.40	0.67

NR, no reconstruction; NSFR, nasoseptal flap reconstruction.

## DISCUSSION

Endoscopic endonasal transsphenoidal surgery is a widely accepted approach for pituitary tumor resections, and its safety and efficacy have been well documented in the literature.^30^–^34^ Nasoseptal flap reconstruction was introduced by Hadad et al. and has come into more common practice as an option for patients presenting large dural defects of the skull base following pituitary surgery.^11^ Assessments of QOL play an important role in evaluating the efficacy of surgical interventions, as the surgeon’s perception of a patient’s QOL has been shown to be inaccurate in the postoperative period.^35^–^37^ This retrospective analysis was conducted to determine the impact of NSFR in endoscopic pituitary adenoma surgery on patients’ long-term QOL via an assessment of SNOT-22 questionnaire responses. In the present study, postoperative (≥12 months) SNOT-22 data were obtained and compared between a group of patients who underwent EETS for pituitary adenoma with NSFR and another group who underwent the same surgery with no reconstruction. The present data showed no statistically significant difference between the groups in overall mean SNOT-22 scores, SNOT-22 subdomain scores, or separate SNOT-22 item scores.

Following harvest of the pedicled NSF, both secretions and blood flow directly onto the exposed cartilage and bone of the septum. The NSF donor site heals by secondary intention and can result in significant crusting for up to 12 weeks postoperatively.^13^,^38^,^39^ De Almeida et al. investigated the time to resolution of nasal crusting, comparing an NSFR cohort to patients who did not have an NSFR.^13^ Patients who had an NSFR did not have a significantly longer time to recovery than patients without an NSFR. In addition, complete remucosalization of the nasal septum after NSFR requires an average 10 to 12 weeks. These data support that there should be no impact on long-term QOL in patients who underwent EETS for pituitary adenoma >1 year from surgery.

In a retrospective study by Pant et al., QOL was measured by Anterior Skull Base (ASB) and SNOT-22 questionnaires. Results revealed a significant improvement in short-term QOL scores for patients who did not undergo NSFR as compared with those who did.^15^ These results may be explained by the fact that patients with larger tumors received NSFR. Moreover, the studied patients had various skull base pathologies, which may have affected research findings. However, limitations of the study design did not allow for conclusions about long-term changes in QOL to be drawn.

In contrast, the present sinonasal QOL study compared two groups with the same pathology and showed no significant differences between the groups. Regarding NSF use, a study by McCoul et al. showed no significant differences in ASB questionnaire scores recorded at 6 months postoperatively. Those findings are compatible with the results of this study,^16^ with the exception that the former did not carry out an assessment of long-term QOL data.

Our study was limited by a relatively small sample size (62 patients). Large-sample studies should be carried out to further evaluate QOL following EETS for pituitary adenoma. Moreover, it should be taken into consideration that there may be some element of selection bias in this study because only those patients who filled out surveys were included in the analysis. Unfortunately, the retrospective nature of this study did not permit a standardized time of administration of postoperative questionnaires.

## CONCLUSIONS

Our results indicate that NSFR in EETS for pituitary adenoma do not mandate poorer long-term postoperative sinonasal QOL outcomes, compared to no reconstruction.

## Abbreviations

EETS: endoscopic endonasal transsphenoidal surgery
CSF: cerebrospinal fluid
NR: no reconstruction
NSF: nasoseptal flap
NSFR: nasoseptal flap reconstruction
QOL: quality of life
SNOT-22: Sinonasal Outcome Test-22.
